# Prevalence and risk factors of preterm birth among pregnant adolescents in Kuala Lumpur: A cross-sectional study

**DOI:** 10.51866/oa.711

**Published:** 2025-09-11

**Authors:** Nur Aznida Abu Bakar, Dalila Roslan, Nik Nairan Abdullah, Musbahiyah Mat Zainal, Pillai Simon Puvanese Rebecca

**Affiliations:** 1 MB BCh BAO, MPH, DrPH, Department of Public Health, Faculty of Medicine, Universiti Teknologi MARA, Sungai Buloh, Selangor, Malaysia. Email: drdalilaroslan@gmail.com; 2 MBBS, MPH, Department of Public Health, Faculty of Medicine, Universiti Teknologi MARA, Sungai Buloh, Selangor, Malaysia.; 3 MBChB, MPH(Family Health), Department of Public Health, Faculty of Medicine, Universiti Teknologi MARA, Sungai Buloh, Selangor, Malaysia.; 4 MBChB, FRACGP, Klinik Kesihatan AU2, Jalan AU2, Taman Seri Keramat, Setiawangsa, Selangor, Malaysia.; 5 MD, MPH, DrPH, Jabatan Kesihatan Wilayah Persekutuan Kuala Lumpur & Putrajaya, Kuala Lumpur, Malaysia.

**Keywords:** Pregnancy in Adolescence, Premature birth, Risk factors, Pregnancy complications

## Abstract

**Introduction:**

Adolescent pregnancies are often associated with a higher risk of adverse outcomes, including preterm birth, which may lead to severe physiological and psychological health impacts on both mothers and children. Despite the acknowledged risks, there is a knowledge gap regarding the risk factors of preterm birth among pregnant adolescents. This study aimed to determine the prevalence and associated sociodemographic, maternal, intrapartum and neonatal factors of preterm birth among pregnant adolescents in Kuala Lumpur.

**Methods:**

A cross-sectional study was conducted in Kuala Lumpur, Malaysia, from March to June 2024. Samples were selected using simple random sampling from the Wilayah Persekutuan Kuala Lumpur and Putrajaya Department of Health pregnant adolescents’ line listing from 2019 to 2023. Simple and multiple logistic regression analyses were used to determine the factors associated with preterm birth.

**Results:**

A total of 175 pregnant adolescents were included in the study. The prevalence of preterm birth was 16%. Anaemia at booking (adjusted odds ratio [aOR]: 7.8; 95% confidence interval [CI]: 2.53, 23.997), history of preterm birth (aOR: 11.654; 95% CI: 1.517, 89.498), history of smoking (aOR: 6.89; 95% CI: 1.397, 34.002) and low birth weight (aOR: 12.503; 95% CI: 3.858, 40.522) were significantly associated with preterm birth.

**Conclusion:**

Targeted interventions to address anaemia, history of smoking, previous history of preterm births and low birth weight babies in adolescent pregnancies are crucial to reduce the incidence of preterm birth and improve maternal and infant health outcomes in this population among adolescents.

## Introduction

Adolescent pregnancy is defined as any pregnancy that occurs in a female individual aged 10-19 years.^1^ Pregnant adolescents are more likely to experience complications such as pre-eclampsia, pregnancy-induced hypertension, anaemia, preterm premature rupture of membrane, and maternal death than adult women.^2^ According to the National Health and Morbidity Survey 2022, the prevalence of adolescent pregnancy in Malaysia was 2.0%.^3^ Although considered low compared to that in other Southeast Asian countries, the prevalence of adolescent pregnancy may be underestimated, as Malaysia did not adequately document data on illegal abortion and abandoned babies among adolescents.^4^ Some of the complications among pregnant adolescents include preterm birth, preeclampsia, maternal anaemia, maternal death and early neonatal demise.^4^

Preterm birth is defined as any birth that occurs before 37 weeks of gestation.^5^ In Malaysia, the rate of preterm birth among pregnant adolescents ranges from 9% to 15.9%.^6,7^ A study conducted in the USA found that adolescents had a significantly higher rate of preterm birth than adults.^8^ Furthermore, preterm birth in adolescent pregnancies inflicts substantial medical, economic and social impacts, as it is associated with acute and chronic complications in both mothers and children.^9,10^ Hence, preterm birth in this particular group may lead to severe physiological and psychological health impacts on both mothers and children, subsequently leading to a poor quality of life.^11^

Despite the acknowledged risk factors of preterm birth in pregnant adolescents such as previous spontaneous preterm birth, low body mass index (BMI), poor gestational weight gain, smoking during pregnancy, premature rupture of membrane, inadequate antenatal care (ANC) and anaemia, there is no local study performed in the primary care setting to assess the prevalence and associated factors of preterm birth in adolescents in an urban setting in Malaysia.^8,12,13^ Early detection of the associated risk factors may enable timely intervention and prevent preterm birth in pregnant adolescents. Therefore, this study aimed to determine the prevalence and associated sociodemographic, maternal, intrapartum, and neonatal factors for preterm birth among pregnant adolescents in Kuala Lumpur.

## Methods

### Study design and participant selection

A cross-sectional study was conducted by reviewing the records of pregnant adolescents who were registered in government health clinics from January 2019 to December 2023. Data were collected from 18 March 2024 to 30 June 2024. Non-Malaysian citizens, pregnant adolescents with multiple pregnancies and pregnant adolescents who were transferred out before giving birth were excluded from the study.

The sample size estimation was calculated using the single-proportion formula in the OpenEpi software. The study power was set at 80%, with an a-value of 0.05, by referring to previous research showing a 9% proportion of preterm birth among pregnant adolescents.^6^ The calculated sample size needed was 126. Given the additional expected 40% missing data, the minimum sample size calculated was 176.^14^ Samples were selected via simple random sampling using the SPSS software.

### Research methodology

**Figure 1:**
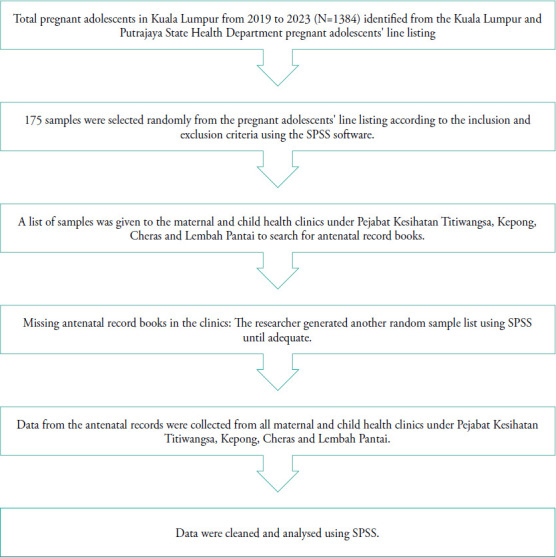
Flowchart of data collection and analysis.

Data were collected using Kuala Lumpur and Putrajaya State Health Department documents from January 2019 to December 2023. Information was extracted from the pregnant adolescents’ line listing from 2019 to 2023 and the maternal antenatal record book KIK 1/(b)/96 from all maternal and child health clinics under Titiwangsa, Kepong, Cheras and Lembah Pantai Health District Offices. Data were reviewed at the respective health department and government health clinic. Figure 1 shows the flowchart of data collection and analysis for this study.

The independent variables extracted from the two data sources consisted of three main components: a) sociodemographic data, b) maternal obstetric data and c) newborn data. The sociodemographic data comprised age, ethnicity, employment status, marital status, educational level and household income. The total household income was categorised into three categories, where B40 represented the bottom 40% of income earners; M40, the middle 40% income earners; and T20, the top 20% income earners in Malaysia.^15^ The total monthly household income of the B40, M40 and T20 groups was less than RM 4999, RM 5000 to RM 9999 and more than RM 10,000, respectively.^16^ The maternal and obstetric data consisted of height, weight and BMI during booking, parity, gestational age at booking, anaemia status at booking, comorbidities, total number of ANC visits, spacing between pregnancies, human immunodeficiency virus (HIV) and venereal disease research laboratory status, history of any miscarriage, history of preterm birth, history of placenta previa, history of premature rupture of membrane, history of lower-segment caesarean section in previous pregnancies, history of any urogenital tract infection, history of vaginal bleeding, history of smoking and vaping, high-risk behaviours and reported psychological issues in the current pregnancy. High-risk behaviours included a history of multiple sexual partners, alcohol abuse and recreational drug abuse. Pregnant adolescents with a haemoglobin level of less than 11.0 g/dL during booking were considered to have anaemia. Those who came for booking after 12 weeks of gestation were classified into the late booking group. Fewer than eight total ANC visits were deemed inadequate. The perinatal and intrapartum data consisted of the mode of delivery and sex and birth weight of the baby. Birth weight was classified as low when it was under 2.5 kg.

The study outcome was preterm birth in pregnant adolescents in Kuala Lumpur. Gestational age during delivery was calculated using Naegele’s rule based on the estimated due date from the line listing.^17^ For participants with unsure or incorrect dates, the gestational age was calculated from the given rectified estimated due date via ultrasound. Those who gave birth before 37 weeks of gestation were classified as having preterm birth.

### Statistical analysis

Data was analysed using by using SPSS Statistic for Windows Version 28.0 (IBM Statistics for Windows, Version 23.0. Armonk, NY: IBM Corp). Descriptive statistics were used to analyse the sociodemographic, maternal, obstetric, intrapartum and neonatal characteristics and the obstetric outcomes of pregnant adolescents. Univariable analysis using simple logistic regression was conducted to identify preliminary factors associated with preterm birth in adolescent pregnancies. From the univariable analysis, statistically significant variables with P-values of <0.25 and clinically significant variables were selected for further analysis. Multivariable logistic regression was utilised to identify the factors associated with preterm birth in pregnant adolescents. The significance level was set at a P-value of <0.05 with a two-tailed test.

## Results

### Sociodemographic, maternal obstetric and newborn data

This study included a total of 175 pregnant adolescents. No missing data were reported. The prevalence of preterm birth among the pregnant adolescents in Kuala Lumpur from 2019 to 2023 was 16% (n=28). Table 1 shows the characteristics of the included pregnant adolescents. The maternal age ranged from 13 to 19 years, with a mean (SD) age of 17.82 (1.272) years. The pregnant adolescents had a mean (SD) height of 1.56 (0.057) m. Most of them came from B40 households (99%) and were pregnant for the first time (77%). The parity of the pregnant adolescents ranged from 0 to 2. The mean (SD) gestational age at booking was 20.85(8.277) weeks, with 81% of the pregnant adolescents coming late for their first antenatal checkup after 12 weeks of gestation based on the perinatal care manual.^18^ None had a history of pre-eclampsia and premature rupture of membrane in their previous pregnancies. No cases of HIV were reported among the participants. Only one participant was reported to be diagnosed with syphilis during the current pregnancy (0.6%). Table 2 presents the participants’ maternal, neonatal and intrapartum characteristics according to their gestational age at delivery.

**Table 1:** Sociodemographic, maternal obstetric and newborn data of the pregnant adolescents.

Variables		Mean (SD)	Frequency, n (%)
Age (year)		17.82 (1.272)	
Weight (kg)		56.65 (13.399)	
Height (m)		1.56 (0.057)	
BMI	Normal		75 (43)
	Underweight		27 (15)
	Overweight		41 (23)
	Obese		32 (18)
Ethnicity	Malay		108 (62)
	Chinese		36 (21)
	Indian		22 (13)
	Others		9 (5)
Marital status	Married		63 (36)
	Not married		112 (64)
Occupational status	Employed		57 (33)
	Unemployed		118 (67)
Household income	B40		173 (99)
	M40		2 (1)
Level of education	No formal education		16 (9)
	Primary education		19 (11)
	Secondary education and above		140 (80)
Parity	Primigravida		135 (77)
	Multigravida		40 (23)
Gestational age at booking (week)		20.85 (8.277)	
Late booking	Yes		142 (81)
	No		33 (19)
Inadequacy of antenatal visits	Yes		48 (27)
	No		127 (73)
Anaemia status during booking	Yes		57 (33)
	No		118 (67)
Spacing between pregnancies	≥2 years		2 (5)
	<2 years		38 (95)
	Primigravida		135 (77)
History of preterm delivery in previous pregnancies	Yes		6 (3)
	No		169 (97)
History of miscarriage/abortion	Yes		15 (9)
	No		160 (91)
History of LSCS in previous pregnancies	Yes		4 (2)
	No		169 (98)
History of pre-eclampsia in previous pregnancies	Yes		0
	No		175 (100)
History of PROM/PPROM in previous pregnancies	Yes		0
	No		175 (100)
HIV status	Positive		0
	Negative		175 (100)
VDRL status	Positive		1 (1)
	Negative		174 (99)
Any urogenital tract infections in the current pregnancy	Yes		32 (18)
	No		143 (82)
Any history of vaginal bleeding during the current pregnancy	Yes		4 (2)
	No		171 (98)
Smoking history (including during pregnancy or before pregnancy)	Yes		18 (10)
	No		157 (90)
High-risk behaviours	Yes		5 (3)
	No		170 (97)
History of any psychological illness	Yes		10 (6)
	No		165 (94)
Comorbidities	Yes		15 (9)
	No		160 (91)
Sex of the baby	Female		92 (53)
	Male		83 (47)
Birth weight of the baby	Low		24 (14)
	Normal		151 (86)
Mode of delivery	SVD		36 (21)
	LSCS/assisted SVD		139 (79)
Preterm birth	Yes		28 (16)
	No		147 (84)

SD: Standard Deviation, BMI: body mass index, LSCS: lower-segment caesarean section, PROM: premature rupture of membrane, PPROM: preterm premature rupture of membrane, HIV: human immunodeficiency virus, VDRL: venereal disease research laboratory, SVD: spontaneous vaginal delivery

**Table 2:** Characteristics of the pregnant adolescents according to their gestational age at delivery.

Variable		Preterm birth (n=28)		Term birth (n=147)		P-value^b^
		Mean (SD)	n(%)	Mean (SD)	n(%)	
Age (year)		17.79 (1.17)		17.83 (1.3)		0.867
Weight (kg)		53.3 (10.6)		57.282 (13.81)		0.15
Height (m)		1.556 (0.073)		1.561 (0.054)		0.347
BMI	Normal		15(53.6)		60(40.8)	0.128
	Underweight		5(17.9)		22(15)	
	Overweight		7(25)		34(23.1)	
	Obese		1(3.6)		31(21.1)	
Ethnicity	Malay		20 (71.4)		88(60)	0.806
	Chinese		4 (14.3)		32(21.8)	
	Indian		3(10.7)		19(12.9)	
	Others		1(3.6)		8(5.4)	
Marital status	Yes		18(64.3)		94(63.9)	1
	No		10(35.7)		53(36.1)	
Occupational status	Employed		21(75)		103 (70.1)	0.658
	Unemployed		7(25)		44(29.9)	
Level of education	No formal education		1(3.6)		15(10.2)	0.25
	Primary education		1(3.6)		18(12.2)	
	Secondary education and above		26(92.9)		114 (77.6)	
Parity	Primigravida		22(78.6)		113 (76.9)	1
	Multigravida		6(21.4)		34(23.1)	
Late booking	Yes		22(78.6)		120 (81.6)	0.792
	No		6(21.4)		27 (18.4)	
Inadequacy of antenatal visits	Yes		17 (60.7)		110 (74.8)	0.164
	No		11(39.3)		37(25.2)	
Anaemia status during booking	Yes		17(60.7)		40(27.2)	<0.001^*^
	No		11(39.3)		107 (72.8)	
History of preterm delivery in previous pregnancies	Yes		3(10.7)		3(2)	0.053
	No		25 (89.3)		144 (98)	
History of miscarriage/abortion	Yes		1(3.6)		14(9.5)	0.338
	No		27(96.4)		133 (90.5)	
History of LSCS in previous pregnancies	Yes		1(3.6)		3(2)	0.51
	No		27(96.4)		142 (96.6)	
Any urogenital tract infections in the current pregnancy	Yes		8(28.6)		24(16.3)	0.179
	No		20(71.4)		123 (83.7)	
Smoking history (including during pregnancy or before pregnancy)	Yes		4(14.3)		14(9.5)	0.496
	No		24(85.7)		133 (90.5)	
High-risk behaviours	Yes		3(10.7)		2(1.4)	0.03^*^
	No		25(89.3)		145 (98.6)	
History of any psychological illness	Yes		1(10.7)		9(6.1)	1
	No		27(96.4)		138 (93.9)	
Comorbidities	Yes		2(7.1)		13(8.8)	1
	No		26(92.9)		134 (91.2)	
Sex of the baby	Female		17(60.7)		75(51)	0.411
	Male		11(39.3)		72(49)	
Birth weight of the baby	Low		12(42.9)		12(8.2)	<0.001^*^
	Normal		16(57.1)		135 (91.8)	
Mode of delivery	SVD		7(25)		29(19.7)	0.61
	LSCS/assisted SVD		21(75)		118(80.3)	

BMI: body mass index, LSCS:lower-segment caesarean section, SVD: spontaneous vaginal delivery

* Statistically significant at α=0.05

b Statistical test: Chi-square and Fisher's exact tests

### Simple logistic regression analysis of the factors associated with preterm birth

The variables with no zero cell values were analysed using simple logistic regression. The significant variables were anaemia status at booking (OR: 4.134; 95% CI: 1.783, 9.585), a history of preterm birth in previous pregnancies (OR: 5.76; 95%CI: 1.13, 30.0163), high-risk behaviours (OR: 8.7; 95% CI: 1.383, 54.717) and low birth weight (OR: 8.437; 95% CI: 3.252, 21.889), with P-values of <0.05. Table 3 shows the results of the simple logistic regression of the independent variables studied.

**Table 3:** Simple logistic regression analysis of the factors of preterm birth among the pregnant adolescents.

Variables		Crude OR (95% CI)	Wald statistic (df)^a^	P-value
Age (year)		0.973(0.711, 1.333)	0.157(1)	0.692
Weight (kg)		0.975 (0.941, 1.01)	2.055(1)	0.152
Height (meter)		0.234(0.0, 307.501)	0.029(1)	0.866
BMI	Normal	REF		
	Underweight	1.037(0.341,3.151)	0.004(1)	0.949
	Overweight	0.76(0.274,2.108)	0.277(1)	0.599
	Obese	0.285(0.035,2.309)	1.383(1)	0.240
Ethnicity	Malay	REF		
	Chinese	0.550(0.175,1.732)	1.043(1)	0.307
	Indian	0.695(0.187,2.577)	0.297(1)	0.586
	Others	0.550(0.065,4.650)	0.301(1)	0.583
Marital status	Married	REF		
	Not married	1.015(0.437,2.358)	0.001(1)	0.973
Occupational status	Unemployed	REF		
	Employed	1.282(0.508,3.233)	0.276(1)	0.599
Level of education	Secondary education and above	REF		
	No formal education	0.292(0.037, 2.313)	1.358(1)	0.244
	Primary education	0.244(0.031, 1.908)	1.809(1)	0.179
Parity	Primigravida	REF		
	Multigravida	1.395	0.497(1)	0.527
Gestational age at booking (week)		0.976(0.929,1.026)	0.880(1)	0.348
Late booking	No	REF		
	Yes	0.825(0.305,2.230)	0.144(1)	0.705
Inadequacy of antenatal visits	No	REF		
	Yes	1.924(0.826,4.478)	2.303(1)	0.129
Anaemia status during booking	No	REF		
	Yes	4.134(1.783,9.585)	10.943(1)	0.001^*^
History of preterm birth in previous pregnancies	No	REF		
	Yes	5.760(1.1,30.163)	4.296(1)	0.038^*^
History of miscarriage/abortion	No	REF		
	Yes	0.352(0.044,2.790)	0.978(1)	0.323
History of LSCS in previous pregnancies	No	REF		
	Yes	1.753(0.176, 17.490)	0.229(1)	0.632
Any urogenital tract infections in the current pregnancy	No	REF		
	Yes	2.050(0.809,5.192)	2.292(1)	0.13
Smoking history	No	REF		
	Yes	1.583(0.480,5.221)	0.57(1)	0.45
High-risk behaviours	No	REF		
	Yes	8.700(1.383, 54.717)	5.317(1)	0.021^*^
History of any psychological illness	No	REF		
	Yes	0.568(0.069,4.669)	0.277(1)	0.599
Comorbidities	No	REF		
	Yes	1.261(0.269,5.923)	0.086(1)	0.769
Sex of the baby	Female	REF		
	Male	0.674(0.296,1.537)	0.879(1)	0.348
Birth weight of the baby	Normal	REF		
	Low	8.437(3.252, 21.889)	19.226(1)	<0.001^*^
Mode of delivery	SVD	REF		
	LSCS/assisted SVD	1.356(0.526, 3.496)	0.398(1)	0.528

REF: reference

* Statistically significant at α=0.05

b Statistical test: Simple logistic regression

### Multivariable analysis

Seven variables from the univariable analysis were included in the multivariable analysis. These variables included ethnicity, level of education, anaemia status, history of preterm birth, history of high-risk behaviours, history of smoking and birth weight of the baby. Four variables were found to be independently associated with preterm birth: anaemia status at booking (adjusted odds ratio [aOR]: 7.8; 95% CI: 2.53, 23.997), history of preterm birth in previous pregnancies (aOR:11.654; 95% CI: 1.517, 89.498), history of smoking (aOR: 6.89; 95% CI: 1.397, 34.002)and low birth weight (aOR: 12.503; 95% CI: 3.858, 40.522) (P<0.05). Table 4 shows the results of the multivariable regression analysis. No significant interactions were observed between the variables mentioned.

**Table 4:** Multivariable logistic regression analysis of the factors associated with preterm birth among the pregnant adolescents.

Variables		Crude OR (95% CI)	aOR (95% CI)	Wald statistic (df)^d^	P-value^c^
Anaemia status during booking	No	REF			
	Yes	4.134(1.783,9.585)	7.8(2.53,23.997)	12.833	<0.001^*^
History of preterm birth in previous pregnancies	No	REF			
	Yes	5.760(1.1,30.163)	11.654(1.517, 89.498)	5.574	**0.018** ^*^
Any smoking history	No	REF			
	Yes	1.583(0.480,5.221)	6.891(1.397,34.002)	5.618	**0.018** ^*^
High-risk behaviours	No	REF			
	Yes	8.700(1.383,54.717)	8.091(0.934,70.091)	3.602	0.058
Birth weight of the baby	No	REF			
	Yes	8.437(3.252,21.889)	12.503(3.858, 40.522)	17.727	**<0.001** ^*^

OR: odds ratio, aOR: adjusted odds ratio, REF: reference

c Backward likelihood ratio test

d Wald test Hosmer—Lemeshow test, P=0.943

* Statistically significant at α=0.05

## Discussion

Pregnancy in adolescents is a significant public health concern in Malaysia. Furthermore, Malaysia is associated with an increased risk of preterm birth, which can have detrimental effects on both the mothers and newborns.^7,19^ In the present study, the prevalence of preterm births among the pregnant adolescents in Kuala Lumpur from 2019 to 2023 was 16%. This prevalence is higher than the reported prevalence in the Malaysian general population (6.63%).^20^ The prevalence in our study is also higher than that reported in another local study conducted in Terengganu, where 9% of pregnant adolescents had preterm birth in 2018.^6^ However, our finding is similar to that of a study performed in a hospital setting in Negeri Sembilan, Malaysia, which showed a 15.9% prevalence of preterm birth in pregnant adolescents from 2015 to 2016.^7^ This might be due to the sociodemographic and population differences between the states.^21^ The prevalence of preterm birth among pregnant adolescents in Malaysia is a cause for concern, as it can lead to various adverse outcomes such as low APGAR scores for babies, fetal anomalies, and stillbirth.^13^

Our study found that anaemia during the initial antenatal visit was associated with an increased risk of preterm birth among pregnant adolescents. This agrees with the findings of a case-control study in Romania, where pregnant adolescents with anaemia had a 35.9% higher risk of preterm births than those without anaemia.^22^ However, a study on the general population in Malaysia also found that mothers with anaemia had higher odds of delivering prematurely.^23^ In Turkiye, mothers with anaemia in the second and third trimesters have been reported to have a higher prevalence of delivering prematurely.^24^ Another previous study discussed that anaemia could induce maternal and fetal stress, which in turn will consequently stimulate the production of the corticotropin-releasing hormone (CRH) and subsequently increase the risk of preterm birth.^25^ Thus, early and aggressive interventions for managing anaemia in pregnant adolescents are crucial to reduce the risk of delivering prematurely. As more than half of the adolescents in this study were found to be unmarried, integrating information in highlighting anaemia as one of the risk factors of preterm birth in adolescent pregnancies in the existing module for the national thalassemia programme in schools is important in preventing preterm birth in this group.

A history of preterm birth in previous pregnancies was found to be a significant risk factor of preterm birth in subsequent pregnancies among the pregnant adolescents in this study. Research has shown that a history of preterm birth is a strong predictor of preterm birth risk in subsequent pregnancies, with the rate of preterm birth increasing in subsequent pregnancies from 22.9% in the second pregnancy to 58.5% in the fourth pregnancy.^26^ Similarly, a study in Thailand found that a history of preterm birth in pregnant adolescents was associated with higher odds of delivering prematurely (OR: 54.42; 95% CI: 16.34, 181.3).^13^ As a history of preterm birth is one of the associated risk factors of preterm birth in the subsequent pregnancy, close monitoring and prenatal care are essential in ensuring adequate care is given to pregnant adolescents.

Smoking during pregnancy is a well-established risk factor of preterm birth. In our study, we found that any history of smoking throughout life was significantly associated with the risk of preterm birth among the pregnant adolescents. In the multivariable analysis, smoking was a significant risk factor of preterm birth, with an aOR of 6.891 (95% CI: 1.397, 34.002). A systematic review concluded that active smoking is associated with increased risks of preterm birth in a dose-response relationship among the general population of France.^27^ The findings in our study can be utilised to justify intensifying efforts to promote anti-tobacco policies, aiming to reduce smoking among the general public and adolescents to create a tobacco-free generation in the future. However, we did not study the duration, frequency or type of tobacco use among the pregnant adolescents due to the limited data available. Hence, we were unable to investigate the correlation between the severity of smoking and the risk of preterm birth in pregnant adolescents, as studied by Delcroix et al. in 2015.^27^

Previous studies have shown that adolescent mothers are at a higher risk of delivering low-birth-weight babies.^6,28^ In this study, we found that having low-birth-weight babies was significantly associated with preterm birth among the pregnant adolescents. Most previous studies have explained that low-birth-weight babies and preterm births are outcomes of adolescent pregnancies^13,28,29^ or that low birth weight is an outcome of preterm birth.^30^ Limited studies have shown that having low-birth-weight babies can cause adolescent mothers to have a higher risk of preterm birth.

Pregnant adolescents’ predisposition to low-birth-weight babies could be attributed to biological factors such as the immaturity of their female reproductive system.^31^ We were unable to identify whether low-birth-weight babies were small for their gestational age. Further studies into the relationship between small-gestational-age babies or low-birth-weight babies and preterm births in adolescent mothers might offer better insights.

Behaviours such as alcoholism, smoking, substance abuse and having multiple sexual partners are classified as high-risk behaviours for pregnancy.^18^ In the univariable analysis of our study, high-risk behaviours were found to be significantly associated with preterm birth among the pregnant adolescents. However, after adjustment, high-risk behaviours were observed to be a confounder for preterm birth. According to a previous study in Thailand, underweight, poor gestational weight gain, a history of premature rupture of membrane and inadequate prenatal care visits are significant risk factors of preterm birth in pregnant adolescents.^13^ However, we were unable to establish these factors as significant risk factors for preterm birth among pregnant adolescents in our study. This is probably due to our small sample size.

Advocating for pre-pregnancy care (PPC) for pregnant adolescents with a history of preterm birth, low-birth-weight babies, smoking and anaemia is crucial to ensure that these adolescents are educated on planning their next pregnancy to reduce the risk of preterm birth. Targeted interventions for high-risk behaviours, such as sexual and reproductive health talks in schools, can reduce adolescent pregnancy and ultimately reduce preterm birth in this population.

### Limitations of the study and recommendations for future study

The main limitation of this study is the use of secondary data. The secondary data available did not include some of the factors that might be of interest, such as health-seeking behaviours, physical activity, stressful life events and domestic violence.^32,33^ A cohort study investigating the risk factors of preterm births in pregnant adolescents would aid in proving the causal relationship between the significant associated risk factors in our study and preterm birth in this population.

## Conclusion

Pregnancy in adolescents can lead to a myriad of health outcomes, including preterm birth. This study depicted an increase in the prevalence of premature birth among pregnant adolescents in Malaysia and showed that premature birth was significantly related to anaemia during booking, history of preterm birth, history of smoking and low birth weight. Targeted interventions to correct iron-deficiency anaemia in pregnancy, reduce smoking and vaping among adolescents and promote close monitoring of adolescent mothers with a history of preterm birth and low-birth-weight babies are essential to prevent preterm birth in this population. PPC or early initiation of ANC for adolescents is important to ensure that they receive adequate ANC and minimise the risk of them delivering prematurely. In view of the scarcity of studies exploring the risk factors associated with preterm birth specifically among pregnant adolescents in a Malaysian primary care setting, the findings of this study may inform the design of future interventions aimed at reducing the incidence of preterm birth in this population.

## Data sharing statement

The data that support the findings of this study are available from the corresponding author, upon reasonable request.

**How does this paper make a difference in general practice?**

- Preterm birth in pregnant adolescents places a more significant burden to both adolescent mothers and the healthcare system.
- Early detection of associated factors may enable timely intervention, such as improved nutrition, management of underlying medical conditions and psychosocial support, potentially preventing preterm birth.
- Understanding the risk factors of preterm birth in pregnant adolescents is imperative to provide insights for developing effective strategies and improving both maternal and neonatal health.
- Significant risk factors, such as anaemia during booking and smoking, can be highlighted in the pink antenatal book as part of screening for red flags specifically for pregnant adolescents.
